# Retrospective analysis of patients with non-tuberculous mycobacteria from a primary hospital in Southeast China

**DOI:** 10.1038/s41598-020-58105-4

**Published:** 2020-01-23

**Authors:** Songjun Ji, Wanping Xu, Jianmin Sun, Yunzhen Shi, Xinling Pan

**Affiliations:** 1 Department of Biomedical Sciences Laboratory, Affiliated Dongyang Hospital of Wenzhou Medical University, Dongyang, Zhejiang China; 2 Department of Clinical Laboratory, Affiliated Dongyang Hospital of Wenzhou Medical University, Dongyang, Zhejiang China; 3 Department of Infectious Diseases, Affiliated Dongyang Hospital of Wenzhou Medical University, Dongyang, Zhejiang China

**Keywords:** Bacterial infection, Infection

## Abstract

To achieve a comprehensive understanding of the characteristics of patients with non-tuberculous mycobacteria (NTM), patients with NTM between January 2016 and June 2019 were recruited from a primary hospital. NTM were identified based on the MBP64 protein assay. The clinical records and laboratory assay results were retrospectively reviewed. A total of 204 patients with NTM were included in the final analysis. The patients with multiple isolations were more likely accompanied with chronic obstructive pulmonary disease (COPD) (p = 0.029) and arthritis (p = 0.049), but showed a lower percentage of positive T-spot results (p = 0.022). In addition, patients with multiple isolations showed a higher rate of positive acid-fast staining results and their symptom duration was more likely longer than 30 days (p = 0.019). Patients with a positive response in T-spot assay showed a higher proportion of nodular manifestation on computed tomography (CT) than those with a negative response. Compared with male patients with NTM, female patients showed lower rates of positive acid-fast staining results (p = 0.03), but were more likely accompanied with COPD (p < 0.0001). The positive acid-fast staining results were closely associated with pulmonary cavities and tuberculosis antibody. Patients with different NTM isolation frequencies were closely associated with coexisting diseases and examination results.

## Introduction

As environmental microbes, non-tuberculous mycobacteria (NTM) can colonize mainly in the lungs and other sites by direct exposure, causing diseases when the balance between the host defense and bacteria is disrupted. The prevalence of NTM diseases has increased in recent years and the 5-year morbidity has been reported to be as high as 17.8% in Korea^1^. Although the clinical significance of NTM isolates remains to be evaluated^2^, NTM diseases cause substantial economic burden^3^.

Given the fact that NTM-related diseases are not mandatory to be treated, patients with NTM are always mis-diagnosed as other chronic diseases such as tuberculosis or cancer^4,5^. Their common symptoms such as cough and expectoration result in difficulties in diagnosis, leading to inappropriate diagnosis and treatment. In fact, in countries with a high incidence rate of tuberculosis such as China, once a patient shows positive results in acid-fast staining assay and T-spot assay, he/she is likely to be diagnosed with tuberculosis and undergo tuberculosis treatment^6,7^. When species identification tests are unavailable or not conducted, patients are not suspected to have NTM infection until they are not susceptible to anti-mycobacterial drugs. Thus, the prevalence and significance of NTM infections are mostly underestimated in China.

Based on the principles of NTM diagnosis proposed by ATS/IDSA, repeated isolation in sputum is required to confirm a pulmonary infection; however, two or more sample collections were mostly unavailable in hospitals in China as NTM surveillance is not mandatory^8^. Sample isolation frequency may help to the discern colonized isolates from the pathogenic ones. Our previous work showed a significant difference between patients with MTBC and those with NTM, but the exact contributions of the isolated NTM to diseases were not determined^9^. The potential difference in patient characteristics for different NTM isolation frequencies remained largely unknown and required to be evaluated urgently^10^.

In this study, the characteristics of patients with NTM were retrospectively analyzed based on the data from a primary hospital located in central Zhejiang Province, in an attempt to determine the potential risk factors associated with NTM diseases. Our results could help clinical staff to comprehensively understand the characteristics of patients with NTM.

## Materials and Methods

The strains were isolated from inpatients admitted at a primary hospital between January 2016 and June 2019. Samples were analyzed via acid-fast staining assay and mycobacterial culture using BACTEC Mycobacterial Growth Indicator Tube 320 systems (BD, USA), simultaneously. When the culture results were positive, the morphology was confirmed by positive acid-fast staining response. Then, the mycobacterial strains were tested via MPB64 protein assay (Hangzhou Genesis, Hangzhou, China) and identified as NTM by a negative response or MTBC by a positive response^11^. The clinical records of inpatients with corresponding NTM isolates were anonymously reviewed and informed consent was obtained from all participants. All experimental protocols were approved by the Ethics Committee of Dongyang People’s Hospital Ethics Committee and Institutional Review Board. All methods were carried out in accordance with relevant guidelines and regulations.

Basic information including gender, age, occupation, smoke history, tuberculosis history, and surgery history was collected. Laboratory examinations included acid-fast staining assay followed by mycobacterial culture, T-spot assay, and tuberculosis antibody assay. The results of computed tomography (CT) of the lung were also reviewed and the presence of nodules or cavities was checked. The coexisting illnesses such as COPD, bronchiectasis, cancer, diabetes, and arthritis were included in the evaluation.

Patients with a single isolation of NTM were referred to as the ones from whom NTM was isolated only once despite the number of samples being sourced and at different intervals at the hospital. Patients with multiple NTM isolations were referred to as the ones from whom NTM was isolated two or more times based on multiple clinical sample sources. If the MPB64 protein assay showed inconsistent results in the samples of patients with multiple isolations, the corresponding patients were excluded from the analysis. When the data for a single patient was found in different years, the latest information was collected and examination results throughout the researched years were reviewed. The suspected or assured diagnosis at discharge was closely associated with later treatment or healthcare; thus, the diagnosis could be classified into tuberculosis, non-tuberculous mycobacterium disease, and non-mycobacterial infection. The duration of symptoms refers to the time from symptom onset to the time when the patient visited the hospital; thus, the symptoms may not be occurring continuously.

### Statistical analysis

All data from the inpatients were categorized into different groups and the differences were analyzed by *X*^2^ test or fisher exact test using 20.0 version SPSS software (IBM, USA). The p values less than 0.05 were considered statistically significant between the analyzed groups.

## Results

A total of 292 NTM strains were identified by MPB64 assay. After excluding repeated samples from the same patients and patients without available clinical records, 204 inpatients were finally analyzed in this study.

Considering that the isolation frequency from sputum was significantly important for the diagnosis of pulmonary NTM, the differences between the patients with single and multiple isolations were analyzed (Table 1). Compared with patients with single isolation, those with multiple NTM isolations showed a significantly higher percentage of surgery history, whereas the two populations were similar in terms of gender, age, occupation, and smoking history. Among the laboratory assay results used for tuberculosis, the patients with multiple isolations showed a significantly higher frequency of positive acid-fast staining results (p = 0.01), but showed a lower percentage of positive T-spot results (p = 0.022). In addition, patients with multiple isolations were more likely accompanied with COPD (p = 0.029) and arthritis (p = 0.049), but not with diabetes, cancer, or bronchiectasis. When discharged from the hospital, significantly more patients with multiple isolations were diagnosed with NTM-related diseases. Finally, patients with a single isolation had experienced the symptoms for a shorter duration (less than 30 days) before visiting the hospital.

**Table 1:** The characteristics of patients with single species and multiple species of non-tuberculosis mycobacteria.

Features	Values	Single isolation (n = 169)	Multiple isolation (n = 35)	*P* value
Gender	Male	94 (55.6)	17 (48.6)	0.446
	Female	75 (44.4)	18 (51.4)	
Age	<60	40 (23.7)	4 (11.4)	0.128*
	60~80	100 (59.2)	27 (77.1)	
	≥80	29 (17.2)	4 (11.4)	
Occupation	Non-farmer	30 (17.8)	4 (11.4)	0.361
	farmer	139 (82.2)	31 (88.6)	
Smoke history	Yes	54 (32.0)	13 (37.1)	0.552
	No	115 (68.0)	22 (62.9)	
Tuberculosis history	Yes	30 (17.8)	9 (25.7)	0.276
	No	139 (82.2)	26 (74.3)	
Surgeon history	Yes	55 (32.5)	21 (60.0))	**0.002**
	No	114 (67.5)	14 (40.0)	
Acid fast assay	Positive	35 (22.4)	18 (51.4)	**0.01**
	Negative	121 (77.6)	17 (48.6))	
CT	Nodular	81 (47.9)	18 (51.4)	0.706
	Non-nodular	88 (52.1)	17 (48.6)	
Cavities	Yes	13 (7.7)	7 (20.0)	0.053*
	No	156 (92.3)	28 (80.0)	
T. spot	Positive	30 (44.8)	3 (15.8))	**0.022**
	Negative	37 (55.2)	16 (84.2))	
Tuberculosis antibody	Positive	20 (15.2)	7 (23.3)	0.278
	Negative	112 (84.8)	23 (76.7)	
COPD	Yes	50 (29.6)	17 (48.6)	**0.029**
	No	119 (70.4)	18 (51.4)	
Bronchiectasis	Yes	39 (23.1)	8 (22.9)	0.978
	No	130 (76.9)	27 (77.1))	
Diabetes	Yes	11 (6.5)	5 (14.3)	0.159*
	No	158 (93.5)	30 (85.7)	
Tumor	Yes	13 (7.7)	3 (8.6)	0.741*
	No	156 (92.3)	32 (91.4)	
Arthritis	Yes	5 (3.0)	4 (11.4)	**0.049**
	No	164 (97.0)	31 (88.6)	
Diagnosis when discharged from hospital	Tuberculosis	49 (30.0)	10 (28.6)	0.005*
	NTM	9 (5.3)	9 (25.7)	
	Non-mycobacterial	111 (65.7)	16 (45.7)	
Duration of symptoms (days)	≥30	89 (52.7)	26 (74.3)	**0.019**
	<30	80 (47.3)	9 (25.7)	

^*^Fisher exact test. The bold values mean significantly with p value less than 0.05.

Because T-spot assay was of significance to distinguish individuals with latent or active TB from healthy individuals, it could also help distinguish between active TB patients and NTM patients among mycobacterial culture-positive patients. The risk factors of a positive T-spot response in patients with NTM are shown in Table 2. Patients with a positive T-spot response showed a higher frequency of nodular manifestations on CT compared with patients with a negative response (p = 0.006); further, a higher proportion of patients with a negative response were aged between 60and 80 years (p = 0.034).

**Table 2:** The characteristics of patients with positive and negative response of T. spot assay.

Features	Values	T. spot Positive(n = 33)	T.spot Negative (n = 53)	P
Gender	Male	23 (69.7)	29 (54.7)	0.167
	Female	10 (30.3)	24 (45.3)	
Age	<60	11 (33.3)	8 (15.1)	**0.034**
	60~80	16 (48.5)	40 (75.5)	
	≥80	6 (18.2)	5 (9.4)	
Occupation	Non-farmer	5 (15.2)	6 (11.3)	0.742*
	Farmer	28 (84.8)	47 (88.7)	
Smoke history	Yes	14 (42.4)	19 (35.8)	0.542
	No	19 (57.6)	34 (64.2)	
Tuberculosis history	Yes	3 (9.1)	13 (24.5)	0.074
	No	30 (90.9)	40 (75.5)	
Surgeon history	Yes	12 (36.4)	22 (41.5)	0.635
	No	21 (63.6)	31 (58.5)	
Acid fast assay	Negative	22 (68.8)	33 (62.3)	0.544
	Positive	10 (31.3)	20 (37.7)	
CT	Nodular	25 (75.8)	24 (45.3)	**0.006**
	Non-nodular	8 (24.2)	29 (54.7)	
Cavities	Yes	3 (9.1)	10 (18.9)	0.354*
	No	30 (90.9)	43 (81.1)	
Tuberculosis antibody	Positive	5 (16.7)	8 (15.1)	1*
	Negative	25 (83.3)	45 (84.9)	
COPD	Yes	7 (21.2)	16 (30.2)	0.36
	No	26 (78.8)	37 (69.8))	
Bronchiectasis	Yes	2 (6.1)	11 (20.8)	0.119*
	No	31 (93.9)	42 (79.2)	
Diabetes	Yes	3 (9.1)	4 (7.5)	1*
	No	30 (90.9)	49 (92.5)	
Tumor	Yes	5 (15.2)	2 (3.8)	0.101^*^
	No	28 (84.8)	51 (96.2)	
Diagnosis when	Tuberculosis	22 (66.7)	16 (30.2)	0.004*
discharged from hospital	NTM	2 (6.1)	9 (17.0)	
	Non-mycobacterial	9 (27.3)	28 (52.8)	
Duration of symptoms (days)	≥30	17 (51.5)	27 (50.9)	1
	<30	16 (48.5)	26 (49.1)	

^*^Fisher exact test. The bold values mean significantly with p value less than 0.05.

Table 3 shows differences between genders in terms of characteristics of patients with NTM. Approximately 20% female patients showed positive acid-fast staining results, which is significantly less compared with the male patients (34.3%; p = 0.03). Moreover, 37.6% of female patients were accompanied with bronchiectasis; this was significantly higher than the percentage in male patients (10.8%, p < 0.0001). The percentage of patients diagnosed with NTM at hospital discharge was higher in males than in females (33.3% vs 5.4%, p < 0.0001).

**Table 3:** The characteristics of patients with non-tuberculous mycobacteria between male and female.

Features	Values	Male (n = 111)	Female (n = 93)	P
Age	<60	22 (19.8)	19 (20.4)	0.728
	60~80	65 (58.6)	58 (62.4)	
	≥80	24 (21.6)	16 (17.2)	
Occupation	Non-farmer	23 (20.7)	11 (11.8)	0.09
	Farmer	88 (79.3)	82 (88.2)	
Smoke history	Yes	66 (59.5)	1 (1.1)	**<0.0001**
	No	45 (40.5)	92 (98.9)	
Tuberculosis history	Yes	23 (20.7)	16 (17.2)	0.525
	No	88 (79.3)	77 (82.8)	
Surgeon history	Yes	39 (35.1)	37 (39.8)	0.494
	No	72 (64.9)	56 (60.2)	
Acid fast assay	Negative	67 (65.7)	71 (79.8)	**0.03**
	Positive	35 (34.3)	18 (20.2)	
Tuberculosis antibody	Positive	17 (19.8)	10 (13.2)	0.26
	Negative	69 (80.2)	66 (86.8)	
CT	Nodular	54 (48.6)	45 (48.4)	0.97
	Non-nodular	57 (51.4)	48 (51.6)	
Cavities	Yes	14 (12.6)	6 (6.5)	0.141
	No	97 (87.4)	87 (93.5)	
COPD	Yes	42 (37.8)	25 (26.9)	0.097
	No	69 (62.2)	68 (73.1)	
Bronchiectasis	Yes	12 (10.8)	35 (37.6)	**<0.0001**
	No	99 (89.2)	58 (62.4)	
Diabetes	Yes	10 (9.0)	6 (6.5)	0.499
	No	101 (91.0)	87 (93.5)	
Tumor	Yes	12 (10.8)	4 (4.3)	0.085
	No	99 (89.2)	89 (95.7)	
Diagnosis when discharged from hospital	Tuberculosis	12 (10.8)	22 (23.6)	**<0.0001**
	NTM	37 (33.3)	5 (5.4)	
	Non-mycobacterial	62 (55.9)	66 (71.0)	
Duration of symptoms (days)	≥30	60 (54.1)	55 (59.1)	0.466
	<30	51 (45.9)	38 (40.9)	

^*^Fisher exact test. The bold values mean significantly with p value less than 0.05.

Imaging manifestations in CT scans were important in the diagnosis of pulmonary infections including pulmonary tuberculosis and NTM diseases. In addition, multiple small nodules were common manifestations of pulmonary MAC diseases. Table 4 shows the characteristics of patients with NTM and nodules. Patients with nodules were slightly younger than those without nodules. Other characteristics including demographic features, laboratory examinations, and coexisting diseases were comparable between these two groups.

**Table 4:** The characteristics of patients with non-tuberculous mycobacteria between nodular and non-nodular.

Features	Values	Nodular (n = 99)	Non-nodular (n = 105)	
Age	<60	28 (28.3)	13 (12.4)	**0.015**
	60~80	52 (52.5)	71 (67.6)	
	≥80	19 (19.2)	21 (20.0)	
Occupation	Non-farmer	15 (15.2)	19 (18.1)	0.573
	Farmer	84 (84.8)	86 (81.9)	
Smoke history	Yes	32 (32.3)	35 (33.3)	0.878
	No	67 (67.7)	70 (66.7)	
Tuberculosis history	Yes	20 (20.2)	19 (18.1)	0.702
	No	79 (79.8)	86 (81.9)	
Surgeon history	Yes	36 (36.4)	40 (38.1)	0.798
	No	63 (63.6)	65 (61.9)	
Acid fast assay	Negative	65 (71.4)	73 (73.0)	0.809
	Positive	26 (28.6)	27 (27.0)	
Tuberculosis antibody	Positive	14 (17.5)	13 (15.9)	0.779
	Negative	66 (82.5)	69 (84.1)	
Cavities	Yes	12 (12.1)	8 (7.6)	0.28
	No	87 (87.9)	97 (92.4)	
COPD	Yes	36 (36.4)	40 (38.1)	0.798
	No	63 (63.4)	65 (61.9)	
Bronchiectasis	Yes	33 (33.3)	26 (24.8)	0.177
	No	66 (66.7)	79 (75.2)	
Diabetes	Yes	8 (8.1)	8 (7.6)	0.902
	No	91 (91.9)	97 (92.4)	
Tumor	Yes	10 (13.1)	6 (5.7)	0.244
	No	89 (86.9)	99 (94.3)	
Diagnosis when discharged from hospital	Tuberculosis	35 (35.4)	24 (22.9)	0.107
	NTM	6 (6.1)	11 (10.5)	
	Non-mycobacterial	58 (58.6)	70 (66.7)	
Duration of symptoms (days)	≥30	55 (55.6)	60 (57.1)	0.819
	<30	44 (44.4)	45 (42.9)	

^*^Fisher exact test. The bold values mean significantly with p value less than 0.05.

Further, the percentage of tuberculosis history, cavities in lung, and positive response in tuberculosis antibody assay was significantly higher in patients with a positive acid-fast staining result than in those with a negative result (Table 5). In addition, patients with positive acid-fast staining results were more likely to be diagnosed with mycobacterial infections including tuberculosis and NTM diseases at hospital discharge.

**Table 5:** The characteristics of patients with non-tuberculous mycobacteria between smear positive and smear positive.

Features	Values	Acid-fast positive (n = 53)	Acid-fast negative (n = 138)	P
Age	<60	12 (22.6)	26 (18.8)	0.42
	60~80	28 (52.8)	87 (63.0)	
	≥80	13 (24.5)	25 (18.1)	
Occupation	Non-farmer	9 (17.0)	23 (16.7)	0.958
	Farmer	44 (83.0)	115 (83.3)	
Smoke history	Yes	19 (35.8)	44 (31.9)	0.602
	No	34 (64.2)	94 (68.1)	
Tuberculosis history	Yes	18 (34.0)	19 (13.8)	**0.002**
	No	35 (66.0)	119 (86.2)	
Surgeon history	Yes	23 (43.4)	50 (36.2)	0.362
	No	30 (56.6)	88 (63.8)	
Tuberculosis antibody	Positive	14 (32.6)	12 (10.5)	**0.001**
	Negative	29 (67.4)	102 (89.5)	
Cavities	Yes	14 (26.4)	5 (3.6)	**<0.001**
	No	39 (73.6)	133 (96.4)	
COPD	Yes	20 (37.7)	46 (33.3)	0.567
	No	33 (62.3)	92 (66.7)	
Bronchiectasis	Yes	14 (26.4)	31 (22.5)	0.564
	No	39 (73.6)	107 (77.5)	
Diabetes	Yes	6 (11.3)	8 (5.8)	0.218*
	No	47 (88.7)	130 (94.2)	
Tumor	Yes	4 (7.5)	10 (7.2)	1*
	No	49 (92.5)	128 (92.8)	
Diagnosis when	Tuberculosis	28 (52.8)	27 (19.6)	<0.001*
discharged from hospital	NTM	13 (24.5)	4 (2.9)	
	Non-mycobacterial	12 (22.6)	107 (77.5)	
Duration of symptoms (days)	≥30	34 (64.2)	78 (56.5)	0.338
	<30	19 (35.8)	60 (43.5)	

^*^Fisher exact test. The bold values mean significantly with p value less than 0.05.

## Discussions

Although the prevalence of NTM increased and its clinical significance in infections begins to be established, a comprehensive understanding of the clinical manifestation and risk factors for NTM infections remain unclear. In this study, the characteristics of inpatients with NTM and the potential risk factors of NTM patients were comprehensively analyzed. We found that the isolation frequency of NTM was associated with laboratory examinations (such as T-spot assay and acid-fast staining assay), coexisting diseases (such as COPD and arthritis), and clinical manifestations (e.g., symptom duration). Further, there were significant differences among patients with NTM in terms of gender, T-spot assay results, and acid-fast staining assay results.

Repeated exposure to NTM in the environment was the main mode of transmission of NTM, and a least of two sputum samples were needed to confirm a diagnosis of pulmonary NTM^8,12^. Moreover, the treatment was not mandatory in patients from whom NTM was isolated, and was determined by the severity of infection as evaluated by the doctors. As reported in a study from Singapore, we found that the patients with more than one isolate were more likely to be accompanied with COPD^10^. A higher percentage of surgery history in multiple isolates suggested that NTM infection may have been hospital acquired^13^. Repeat isolation of NTM from the same patient provided valuable information for the diagnosis of NTM diseases and suggest a persistent infection (duration of symptoms longer than 30 days in this study). There is a huge data to suggest that chronicity of several NTMs is associated with genetic defects in IFN expression. The patients with NTM infection more likely showed defect in signal transduction of IFN-γ/IL-12 axis, especially in patients with disseminated NTM diseases^14^. Besides, the patients with NTM infection commonly express high levels of anti-IFN-γ autoantibodies and suffer from recurrent infection^15,16^.

NTM was more likely to infect older females as reported previously^1,17,18^; this may suggest that estrogen has a protective role against NTM^18^. However, in other studies including our study, no significant difference of distribution was found between genders^2,10^, and the prevalence of NTM in younger individuals should also be given attention^6^. The difference of prevalence between genders may be due to the heterogenicity of the infection-causing microorganism among different regions^2^ or the distribution of ages between genders^19^. In our study, female patients with NTM showed a significantly higher percentage of bronchiectasis than male patients, similar to the results reported by Zhang et al in Singapore^10^. In addition, authors from the same study found that a higher proportion of male patients had COPD, but this difference was not significant in our study^10^. Although several studies have shown that structural lung diseases such as COPD and bronchiectasis predispose people to NTM, the gender may play a potential role in determining the risk of coexisting diseases in patients with NTM. This phenomenon is urgently needed to be proven in a larger population and the mechanisms should be explored in the future.

The laboratory examinations for NTM were limited. For the diagnosis of MAC diseases, the potential of anti-GPL (glycopeptidolipid) IgA in the serum has been researched and has indicated encouraging results in terms of specificity and sensitivity^20,21^. However, considering the diversity in species composition and largely unknown epidemiology of NTM in China, diagnosis using anti-GPL IgA needs to be investigated in the future. The molecular identification including genes (rpoB, hsp65, 16S rRNA and ITS region) sequencing rely on a large number of the strains, and should be performed based on a positive culture^22–24^. LPA and GeneXpert assays (detecting MTB/RIF) could differentiate MTB and NTM with considerable specificity and sensitivity in a broad range of sample sources, but it has not been applied commonly in primary hospitals because of requiring special equipment^25^. Thus, the acid-fast staining assay of samples before culture and immunological assays such as IFN-gamma release assay and T-spot assay would provide timely information for the diagnosis of active or latent tuberculosis^26,27^. However, patients with NTM have shown a high proportion of positive T-spot results in our previous study and another study^9,28^. In this study, a positive T-spot assay result in patients with NTM was associated with nodular manifestation in CT, indicating that a potential relation between nodule formation and immunological response may exist in these patients. In addition, positive results in acid-fast staining assay in patients with NTM suggest a large amount of isolates in individuals, resulting in a severe response in pulmonary infections, shown as cavities in the lung^29,30^. The risk factors determined in laboratory examinations would help us evaluate the progress of infection in patients with NTM. The limitations of this study include the possibility of colonization and infection both in patients with single isolations. This leads to a difficulty in establishing an association between the isolated strains and the diseases the patient suffers; however, this is a common phenomenon in China where NTM infections have not been paid enough attention, especially in primary hospitals and rural regions. In the future, patients from whom NTM strains are isolated should be recruited and checked to determine whether the isolates exist persistently.

## Conclusions

Although the NTM related infection has been increasingly reported worldwide, most NTM cases are derived from the patients suspected of tuberculosis^31,32^. Routine diagnosis of tuberculosis is inadequate for accurate diagnosis of NTM infection, which leads to underestimated prevalence^33,34^. When molecular methods for species identification are non-available in resources limited region, comprehensive analysis of patients with NTM strains will shed light on realizing the importance of NTM infections in primary hospitals. In addition, laboratory examination results are closely associated with clinical or radiological manifestations in this study, indicating a comprehensive analysis of risk factors was necessary in diagnosis of NTM related diseases.
